# Biomechanical Analysis of Force Distribution in Human Finger Extensor Mechanisms

**DOI:** 10.1155/2014/743460

**Published:** 2014-07-09

**Authors:** Dan Hu, Lei Ren, David Howard, Changfu Zong

**Affiliations:** ^1^State Key Laboratory of Automotive Simulation and Control, Jilin University, Changchun 130022, China; ^2^School of Mechanical, Aerospace and Civil Engineering, University of Manchester, Manchester M13 9PL, UK; ^3^Key Laboratory of Bionic Engineering of Ministry of Education, Jilin University, Changchun 130022, China; ^4^School of Computing, Science and Engineering, University of Salford, Manchester M5 4WT, UK

## Abstract

The complexities of the function and structure of human fingers have long been recognised. The in vivo forces in the human finger tendon network during different activities are critical information for clinical diagnosis, surgical treatment, prosthetic finger design, and biomimetic hand development. In this study, we propose a novel method for in vivo force estimation for the finger tendon network by combining a three-dimensional motion analysis technique and a novel biomechanical tendon network model. The extensor mechanism of a human index finger is represented by an interconnected tendinous network moving around the phalanx's dorsum. A novel analytical approach based on the “Principle of Minimum Total Potential Energy” is used to calculate the forces and deformations throughout the tendon network of the extensor mechanism when subjected to an external load and with the finger posture defined by measurement data. The predicted deformations and forces in the tendon network are in broad agreement with the results obtained by previous experimental in vitro studies. The proposed methodology provides a promising tool for investigating the biomechanical function of complex interconnected tendon networks in vivo.

## 1. Introduction

The complexities of the function and structure of human finger have long been recognised [1–4]. Effective function of the finger requires the stability and strength made possible by coordinated musculotendon actions subject to the constraining forces exerted by joint capsules, ligaments, and joint articular surfaces. In manual activities, the highly complex musculoskeletal system of the hand and forearm is extremely well coordinated to generate appropriate fingertip forces and finger postures. Assessment of the forces transmitted by the musculotendon complexes and the various joints not only contributes to our understanding of normal human function and the etiology of hand diseases, but may also significantly improve prosthetic and biomimetic hand design.

Over recent decades, a large number of biomechanical studies have been conducted to investigate the forces and moments transmitted in the musculoskeletal system of the human finger using analytical functional representations of the tendon network, which are all based on the conventional equilibrium method and do not explicitly represent the tendon network's geometry [5–12]. Although these functional models can provide useful information on the mechanical and physiological state of human fingers, computational analyses of the finger tendon network that take proper account of its complex geometry are important for studying the physical interactions between the different components of the tendon network [13–17]. The loading of each tendon component within the extensor mechanism should be quantified in order to comprehend the complex temporal and spatial coordination patterns of the multiple musculotendon units involved in finger movements. In addition, as many finger injuries involve rupture of the connective tissues of the extensor mechanism, information about the loading of each component in the tendon network may help us to better understand injury mechanisms.

Therefore, in this study, we developed a novel method to assess the in vivo forces in the finger extensor mechanism by combining a three-dimensional motion analysis technique and a novel biomechanical tendon network model. The finger extensor mechanism is modelled using an interconnected tendinous network moving around the phalanx's dorsum. A novel analytical approach based on the “Principle of Minimum Total Potential Energy” is used to calculate the forces and deformations throughout the tendon network of the extensor mechanism when subjected to a measured external load and finger posture.

## 2. Methods

### 2.1. Tendon Network Modelling

**Figure 1:**
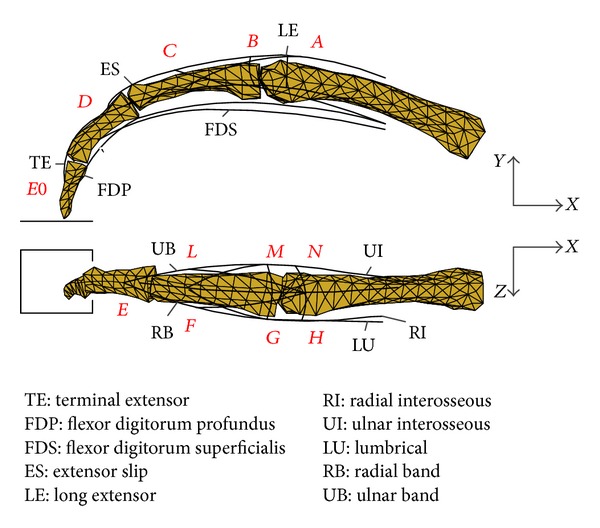
A three-dimensional multisegment model of the human index finger with four segments and three joints (lateral and superior views).

In this study, the finger extensor mechanism is modelled as an interconnected tendon network moving over the phalanges (see Figure 1). Each individual tendon component is represented as a linear spring that can only transmit force in the taut state. Hence its stiffness *K*_*i*_ satisfies the following conditions:

(1) $$\begin{array}{c} k_{i}\left(l\right)=\{\begin{array}{cc} K_{i} & \text{if}\;\;l_{i}\geq {l_{i}}_{0}\\ 0 & \text{if}\;\;l_{i}<l_{i0}, \end{array} \end{array}$$

where *K*_*i*_ is the spring stiffness determined by the material characteristic of the tendon, *l*_*i*_ is the length of the individual tendon component, and *l*_*i*0_ is the slack length of the tendon component. The length *l*_*i*_ is determined by the tendon component's path, over the bone surface, between two of the tendon network nodes (e.g., nodes *A* and *B* in Figure 1). Based on the stiffness defined in (1), the tendon force *F*_*i*_ can be simply related to its length change as follows:

(2) $$\begin{array}{c} F_{i}=k_{i}\left(l\right)\cdot \left(l_{i}-l_{i0}\right). \end{array}$$

In order to determine the positions of the nodes of the tendon network and, hence, the forces in the individual components of the tendon network, the Principle of Minimum Total Potential Energy is used. This is done by minimising the system's total potential energy Π, which is the sum of the strain energy stored in the system, *U*, and the potential energy of the external forces, *V*, which corresponds to

(3) $$\begin{array}{c} \delta \prod =\delta \left(U+V\right)=0. \end{array}$$

**Figure 2:**
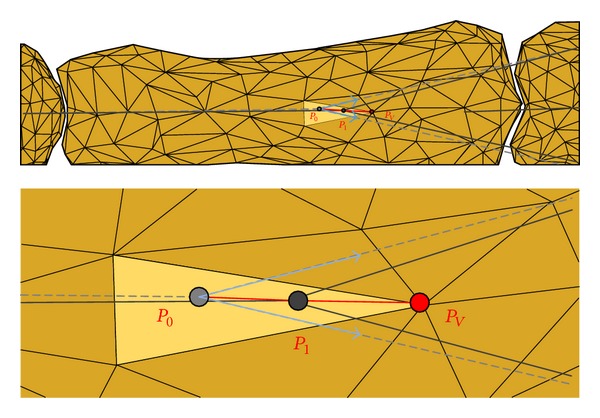
The movement constraint applied to each node in the tendon network. Each node is constrained to move along the line between the node itself (*P*_0_) and one vertex (*P*_*V*_) of the bone facet containing the node to a new position (*P*_1_). The vertex chosen is the one which leads to the direction of node movement being closest to the direction of the tendon force.

By applying this principle to the tendon network, the following set of differential equations was obtained with respect to the coordinates of the nodes that define the 3D geometry of the interconnected network (see Figure 1):

(4) ∂(∑i=1m(ki(li)·(li−li0)2/2)−∑i=1nFi·si)∂pjk=0(j=1,2,3;k=1,2,…,l),

where *s*_*i*_ is the displacement of the tendon component, and *p*_*jk*_ is the *j*th coordinate of the *k*th node of the tendon network. By solving these differential equations, the nodes' coordinates in 3D space were calculated. During manual activities, the tendon components wrap around the bone surfaces of the phalanges; thus, additional constraints are needed to define the spatial path of each tendon component. In this study, we simplify each tendon component's path by assuming it follows a straight line between two nodes in the tendon network. The nodes are constrained to be on the bone surface and to move along the line between the node itself and one particular vertex of the bone facet containing the node. The vertex chosen is the one which leads to the direction of node movement being closest to the direction of the tendon force (see Figure 2). Thereafter, the Lagrange multiplier method is used in combination with (3) to minimise the total potential energy, subject to satisfying the* “node stays on bone surface” *constraint. The minimisation problem and the corresponding Lagrange function are defined in (5), where *L*_*s*_(*p*_*jk*_) = 0 is the equality constraint that* “keeps the node on the line between the node itself and a vertex,”* and *λ*_*s*_ is the Lagrange multiplier:

 Minimize Π(*p*_*jk*_)
 Subject to *L*_*s*_(*p*_*jk*_) = 0
  (5) $$\begin{array}{c} \Lambda \left(p_{jk},\lambda_{s}\right)=\Pi \left(p_{jk}\right)+\overset{r}{\underset{s=1}{\sum }}\lambda_{s}L_{s}\left(p_{jk}\right). \end{array}$$

This leads to the following set of equations, which are used to solve for the coordinates of the nodes in the tendon network:

(6) (∂(∑i=1m(ki(li)·(li−li0)2/2)  −∑i=1nFi·si+∑s=1rλsLs(pjk)))(∂pjk)−1=0(∂(∑i=1m(ki(li)·(li−li0)2/2)  −∑i=1nFi·si+∑s=1rλsLs(pjk)))(∂λs)−1=0(j=1,2,3;k=1,2,…,l).

### 2.2. Multisegment Finger Model

**Figure 3:**
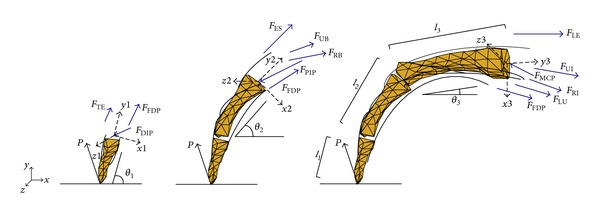
Free body diagrams for the multisegment finger model at each joint during static pressing.

The external loads applied to the tendon network acted along the lines representing the muscles' RI, LU, LE, and UI. To provide kinematic and kinetic inputs for the tendon network model, a multisegment finger model was constructed (Figure 3). Considering moment equilibrium around each DoF of the three joints, we get

(7) FFDPaFDP_DIP_FL−FTEaTE_DIP_FL  +Pxl1sinθ1−Pyl1cos⁡θ1=0,FFDPaFDP_PIP_FL−FESaES_PIP_FL−FUBaUB_PIP_FL  −FRBaRB_PIP_FL+Px(l1sinθ1+l2sinθ2)  −Py(l1cos⁡θ1+l2cos⁡θ2)=0,FFDPaFDP_MCP_FL−FLEaLE_MCP_FL+FRIaRI_MCP_FL  +FUIaUI_MCP_FL+FLUaLU_MCP_FL  +Px(l1sinθ1+l2sinθ2+l3sinθ3)  −Py(l1cos⁡θ1+l2cos⁡θ2+l3cos⁡θ3)=0,FRIaRI_MCP_AD−FUIaUI_MCP_AD+FLUaLU_MCP_AD  +Pz(l1cos⁡θ1+l2cos⁡θ2+l3cos⁡θ3)=0,

where *F*_TE_, *F*_ES_, *F*_RB_, *F*_UB_, *F*_LE_, *F*_UI_, *F*_RI_, and *F*_LU_ are the forces exerted by the major muscles and tendons of the finger extensor mechanism. *F*_FDP_ is the force of the finger flexor muscle that spans the three joints. The force of the other flexor muscle FDS has been merged with *F*_FDP_ due to its relatively small contribution to index finger pinching [18]. *a*_FDP_DIP_FL_ and *a*_TE_DIP_FL_ are the flexion/extension moment arms around the DIP joint. *a*_ES_PIP_FL_, *a*_UB_PIP_FL_, and *a*_RB_PIP_FL_ are the flexion/extension moment arms around the PIP joint. *a*_LE_MCP_FL_, *a*_RI_MCP_FL_, *a*_UI_MCP_FL_, and *a*_LU_MCP_FL_ are the flexion/extension moment arms around the MCP joint. *a*_RI_MCP_AD_, *a*_UI_MCP_AD_, and *a*_LU_MCP_AD_ are the adduction/abduction moment arms around MCP joint. The external load *P* exerted at the fingertip during pressing, the angles of the phalanx segments (*θ*_1_, *θ*_2_, *θ*_3_) with respect to the *X* axis of the global coordinate system (see Figure 3), and also the phalangeal lengths (*l*_1_, *l*_2_, *l*_3_) are determined from measurement data (see following section).

In order to solve this statically indeterminate problem, the following set of validated constraint equations based on an in vitro anatomical study was used [18–20]:

(8) $$\begin{array}{c} F_{\text{RB}}=\frac{2}{3F_{\text{LU}}}+\frac{1}{6F_{\text{LE}}},\\ F_{\text{UB}}=\frac{1}{3F_{\text{UI}}}+\frac{1}{6F_{\text{LE}}},\\ F_{\text{TE}}=F_{\text{RB}}+F_{\text{UB}},\\ F_{\text{ES}}=\frac{1}{3F_{\text{RI}}}+\frac{1}{3F_{\text{UI}}}+\frac{1}{3F_{\text{LU}}}+\frac{1}{6F_{\text{LE}}},\\ F_{\text{LE}}=F_{\text{ES}}. \end{array}$$

The calculated muscle forces *F*_RI_, *F*_LU_, *F*_LE_, and *F*_UI_ are the external forces applied to the finger tendon network at nodes *H*, *G*, *A*, and *N*, respectively. Then, after all of the tendon component forces have been obtained by minimising the total potential energy, the bone-to-bone contact forces *F*_DIP_, *F*_PIP_, and *F*_MCP_ are calculated from the force equilibrium for each joint. The entire biomechanical analysis described above was implemented in the Matlab programming environment (Mathworks, MA, USA).

### 2.3. Measurement of Static Finger Pressing

**Figure 4:**
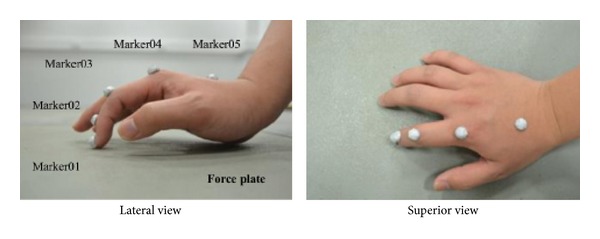
Experimental setup for the measurement of the 3D fingertip force and finger posture during maximum isometric pressing.

**Figure 5:**
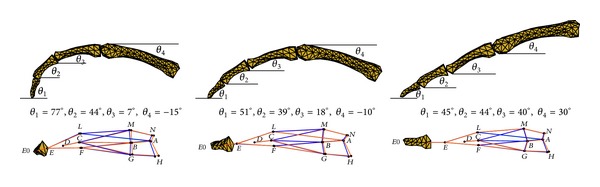
Three-dimensional deformation of the tendon network at the three different finger pressing postures. The blue lines show the unloaded positions, whereas the red lines represent the deformed positions after the load is applied.

The 3D finger postures and the external forces at the fingertip during static pressing by one healthy male subject (age: 25, weight: 75 kg, height: 1.72 m) were measured to provide kinematic and kinetic inputs for the finger model. The subject provided informed consent in accordance with the policies of the institute's ethical advisory committee. The subject was instructed to press the force plate surface using his index finger for approximately 3 seconds with maximum voluntary isometric force. Static pressing measurements were taken at three different finger postures, from very flexed to fully extended (see Figure 5). Each experimental condition was measured ten times. Motion data were recorded at 200 Hz using a six-camera motion analysis system (Vicon, Oxford, UK). Two force plates (Kistler, Switzerland) were used to record fingertip forces at 1000 Hz. Five semireflective markers of 8 mm diameter were attached on the distal phalanx dorsal head (Marker01), middle phalanx dorsal head (Marker02), proximal phalanx dorsal head (Marker03), metacarpal bone dorsal head (Marker04), and metacarpal bone dorsal base (Marker05) to capture finger motion (see Figure 4).

The raw marker data was processed using customised Matlab codes. All trials with more than 10 consecutive missing frames were discarded. After fill-gap processing, the data were filtered using a low-pass zero-lag fourth-order Butterworth digital filter with a cut-off frequency of 6.0 Hz. For both marker and force plate records, only the data in the middle of the trial when the subject had reached a steady isometric pressing condition were used. After data processing, the measured external load *P* and the phalanx angles *θ*_1_, *θ*_2_, *θ*_3_, and *θ*_4_ at a representative instant of time were used as inputs to the multisegment finger model and the tendon network model for in vivo force calculation.

## 3. Results

The measured 3D fingertip forces and positions of the phalanges from three representative static pressing trials, each for a different finger posture varying from a flexed position to a fully extended position (see Figure 5), were used as the model inputs. Using the muscle forces estimated by the multisegment finger model, the tendon network model was then used to calculate the forces transmitted along each tendon component. Thereafter, the bone-to-bone contact forces at each joint were calculated.

Table 1 lists the measured fingertip forces (*P*_*x*_, *P*_*y*_, *P*_*z*_) and the phalanx angles (*θ*_1_, *θ*_2_, *θ*_3_, *θ*_4_) for each of the three finger postures. The calculated muscle forces for the four major finger muscles (RI, LU, LE, and UI) and also the bone-to-bone contact forces at each finger joint during pressing are listed in Table 1. It can be seen that the force magnitudes for the four major finger muscles range from 0.1 to 3.9 times the applied fingertip load, which is in broad agreement with the averaged force data for isometric pinching reported by Chao et al. [18]. Also, the predicted bone-to-bone contact forces at the DIP and PIP joints range from 8.0 to 14.6 times the applied load, which is in broad agreement with the data presented by An et al. [21]. However, the bone-to-bone contact force at the MCP joint varies between 9.1 and 13.8 times the applied load, which is lower than the range (14.7–27.1) reported by An et al. [21]. This is probably because, in their analysis of the extensor mechanism, they neglected the tendon network, which may contribute to the attenuation of the MCP joint contact forces. Generally, the calculated muscle forces decrease with a more extended finger posture, which agrees well with the trends seen in previous studies [6, 22].

Figure 5 shows the 3D deformation of the tendon network during static fingertip pressing for the three different postures. It can be seen that the 3D geometry of the entire tendon network undergoes small deformations due to the pulling of the extensor muscles. The shape change of the network becomes more noticeable when the finger becomes more flexed. This is consistent with the larger muscle forces predicted in the more flexed finger posture.

**Figure 6:**
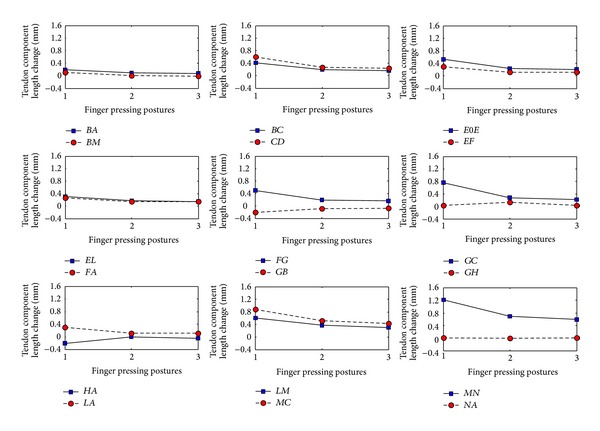
Predicted length changes of the tendon components during maximum isometric pressing at three different postures. Posture 1 is the least extended and posture 3 is the most extended.

**Figure 7:**
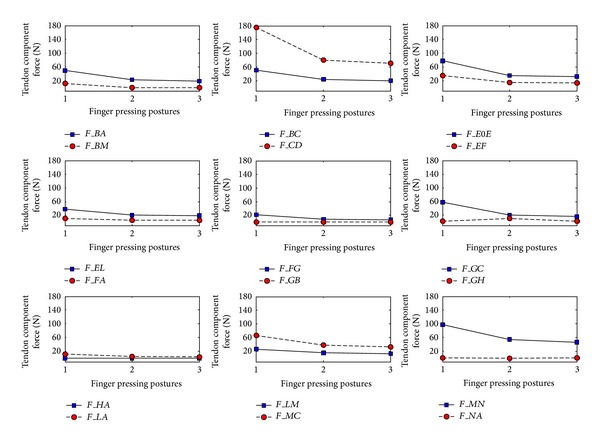
Predicted tendon forces of the tendon components during maximum isometric pressing at three different postures. Posture 1 is the least extended and posture 3 is the most extended.

Figures 6 and 7 show the changes in tendon component lengths and the tendon component forces, respectively, for all tendon components in the extensor mechanism and for all three postures. It can be seen that, for most of the tendon components, both tendon length and force decrease appreciably with a more extended pressing posture. The calculated values of length change and force for each tendon component for the three different postures are also listed in Tables 3(a), 2, and 3(b). From the tables, it is apparent that the tendon component* MN* experiences the maximum tendon length change for all three postures. This is probably due to the action of the major extensor muscle* UI*, which generated the maximum force among the four finger extensor muscles (see Table 1). Although the tendon component* CD* produces the largest tendon force, it does not experience the maximum length change as it possesses a much higher stiffness than the* MN* component [23]. It is noteworthy that the predicted length changes of the tendon components are all well within the ranges found in a previous in vitro experimental study using 7 cadaveric hands by Garcia-Elias et al. [24]. Furthermore, the fact that the* CD* component produces the maximum tendon force while the cross tendon components* FA* and* LA* as well as the lateral components* FG* and* LM* sustain smaller forces agrees well with the results obtained by Valero-Cuevas and Lipson [13].

## 4. Discussion 

The extensor mechanism of the human finger is a highly complicated musculoskeletal system with a complex assembly of multidirectional components with different viscoelastic properties. Although the anatomical structure and mechanical properties of the extensor mechanism have been investigated in several in vitro experimental studies [23, 24], quantifying the tendon force distribution remains challenging. In this study, we have proposed a novel biomechanical musculoskeletal model to estimate the in vivo force distribution within the finger extensor tendon network by combining a 3D motion analysis technique and a novel biomechanical tendon network model. The predicted deformations and forces in the extensor tendon network broadly agree with the results obtained by previous experimental in vitro studies. As well as estimating the forces generated by each tendon component, the proposed method can also be used to calculate the bone-to-bone contact forces at each finger joint. Thus, it can be used to provide comprehensive biomechanical data for in vivo loading of the musculoskeletal complex of the human finger.

The forces transmitted in the extensor tendon network were calculated using the Principle of Minimal Total Potential Energy. This energy-based formulation provides an effective method for analytically investigating the force distribution in complex tendon networks. Unlike the conventional method, which uses the equilibrium equations, no additional compatibility equations are needed to solve the force distribution in the tendon network, which is highly indeterminate statically. In comparison to the relaxation method proposed by Valero-Cuevas et al. [13, 14, 16], the approach proposed here is more computationally efficient because it uses a closed-form analytical method to solve the tendon force distribution problem and no numerical iteration is needed as is the case for the relaxation method.

To predict the in vivo muscle forces during fingertip pressing, a set of well-established empirical equations (see (8)) obtained from a previous in vitro experimental study were used to resolve the redundancy problem resulting from static indeterminacy. Although many alternative methods could be employed to solve this force redundancy problem, such as linear programming or numerical optimisation, and so forth [9, 10, 25, 26], the major aim of this study was to propose a novel energy-based approach to tendon network modelling but not to present a new method for solving the redundancy problem.

To simplify the modelling process, some noteworthy assumptions were employed in the multisegment finger model and in the tendon network model. The tendons are represented as line springs (for stiffness values, see [23]) that can only generate force in lengthening, rather than as 3D solid fibres. In addition, the tendon network is considered to be a simple structure formed by straight lines via connecting nodes. Among the nodes, only* E0* and *D* attach to the bone while the other nodes move around the bone surfaces. In this study, some rather simple constraint equations are used to define the interaction between tendons and bones. The nodes of the tendon network are constrained to only move on the lines containing vertexes of the 3D surfaces of phalanx's bones. Constraint equations were defined based on the geometry of the facet in contact with the tendon component. However, practically, the nodes can move around the entire surface of the contact area rather than move along some particular predefined lines on the surface. In addition, only nodes being constrained to move on the bone surface are not enough. Therefore, an effective bone wrapping algorithm is needed to further improve the tendon network model by representing more realistic geometrical changes of the tendon components whilst moving around bones.

Although the extensor mechanism of a single index finger was modelled in this study, the proposed energy-based method can be applied to the other human fingers and to the whole human hand complex as well. This would allow us to predict the structure deformation and the force distribution of the finger extensor mechanisms at different hand activities, for instance, precision grip [17] or finger pressing [27]. The revealed force transmission pattern in the musculotendon network of the human hand complex would help us to gain better understanding of the functional anatomy and biomechanical functions of the human hand structure.

A subject-specific experimental validation of the predicted deformations and forces in the extensor tendon network appears impossible at present due to the limitations of the current measurement techniques available to quantify the internal mechanical state of the musculoskeletal system in vivo. However, good agreement with in vitro experimental studies in terms of the general trends and the ranges of the predicted deformations and forces in the extensor tendon network suggest that the proposed method is a promising tool for investigating the in vivo biomechanics of complex interconnected musculoskeletal systems.

## 5. Conclusions

In this study, we proposed a novel method for in vivo force evaluation of the finger tendon network by integrating the three-dimensional motion analysis technique and a novel biomechanical tendon network model. The Principle of Minimum Total Potential Energy is used to assess the force transmissions throughout the tendon network of the extensor mechanism when subjected to the external load and the finger posture defined by the measured data. The proposed method can not only evaluate the forces acting on each individual tendon components, but also can work out the bone-to-bone contact forces at each finger joint and, hence, may provide a comprehensive biomechanical database of the in vivo loading condition of the human finger musculoskeletal complex.

## Figures and Tables

**Table 1:** The measured pressing load, the phalanx angles, the calculated extensor muscle forces (normalised by pressing load), and the bone-bone contact forces (normalised by pressing load) under maximum isometric finger pressing at three different postures.

Phalanx angle (degree)				Press load *P* (N)			Extensor muscle force				Bone-to-bone contact force		
*θ*_1_	*θ*_2_	*θ*_3_	*θ*_4_	*P*_*x*_	*P*_*y*_	*P*_*z*_	*F*_RI_	*F*_LU_	*F*_LE_	*F*_UI_	*F*_DIP_	*F*_PIP_	*F*_MCP_
77	44	4	−15	−0.95	25.48	0.90	0.1*P*	3.1*P*	2.8*P*	3.9*P*	9.7*P*	14.6*P*	13.8*P*
51	39	18	−10	2.05	25.20	1.18	0.4*P*	0.7*P*	1.3*P*	2.2*P*	8.0*P*	10.4*P*	10.2*P*
45	44	40	30	6.16	25.29	1.02	0.1*P*	0.8*P*	1.1*P*	1.9*P*	8.3*P*	10.3*P*	9.1*P*

**Table 2:** The predicted length changes and tendon forces for each tendon component of the extensor mechanism during maximum isometric finger pressing at posture 2.

Tendon component	Length change (mm)	Force (N)
*E0E *	0.237	35.47
*AB *	0.090	23.09
*BC *	0.191	24.07
*CD *	0.0271	79.81
*EF *	0.0121	14.61
*FG *	0.0186	8.00
*GH *	0.0132	10.58
*GB *	−0.0081	0
*HA *	0.0006	0.07
*EL *	0.170	20.52
*LM *	0.373	16.03
*MN *	0.688	55.07
*MB *	−0.004	0
*NA *	0.002	0.21
*FA *	0.151	6.05
*GC *	0.276	20.68
*LA *	0.122	4.86
*MC *	0.514	38.53

**Table:** (a)

Tendon component	Length change (mm)	Force (N)
*E0E *	0.521	35.47
*AB *	0.191	48.87
*BC *	0.409	51.55
*CD *	0.595	174.85
*EF *	0.289	34.97
*FG *	0.491	21.11
*GH *	0.033	2.64
*GB *	−0.210	0
*HA *	−0.211	0
*EL *	0.313	37.85
*LM *	0.612	26.32
*MN *	1.217	97.37
*MB *	0.102	12.35
*NA *	0.008	0.93
*FA *	0.271	10.85
*GC *	0.766	57.42
*LA *	0.307	12.27
*MC *	0.887	66.55

**Table:** (b)

Tendon component	Length change (mm)	Force (N)
*E0E *	0.212	31.77
*AB *	0.075	19.14
*BC *	0.158	19.89
*CD *	0.240	70.45
*EF *	0.112	13.60
*FG *	0.158	6.81
*GH *	0.036	2.84
*GB *	−0.069	0
*HA *	−0.043	0
*EL *	0.154	18.58
*LM *	0.303	13.01
*MN *	0.590	47.17
*MB *	−0.018	0
*NA *	0.007	0.90
*FA *	0.015	5.92
*GC *	0.224	16.78
*LA *	0.109	4.37
*MC *	0.438	32.84

## Notation

FDP: Flexor digitorum profundus
FDS: Flexor digitorum superficialis
TE: Terminal extensor
ES: Extensor slip
LE: Long extensor
RI: Radial interosseous
UI: Ulnar interosseous
LU: Lumbrical
RB: Radial band
UB: Ulnar band
DIP: Distal interphalangeal
PIP: Proximal interphalangeal
MCP: Metacarpophalangeal
*a*_TE_DIP_FL_: Flexion/extension moment arm of TE around DIP joint
*a*_FDP_DIP_FL_: Flexion/extension moment arm of FDP around DIP joint
*a*_ES_PIP_FL_: Flexion/extension moment arm of ES around PIP joint
*a*_UB_PIP_FL_: Flexion/extension moment arm of UB around PIP joint
*a*_RB_PIP_FL_: Flexion/extension moment arm of RB around PIP joint
*a*_FDP_PIP_FL_: Flexion/extension moment arm of FDP around PIP joint
*a*_LE_MCP_FL_: Flexion/extension moment arm of LE around MCP joint
*a*_RI_MCP_FL_: Flexion/extension moment arm of RI around MCP joint
*a*_UI_MCP_FL_: Flexion/extension moment arm of UI around MCP joint
*a*_LU_MCP_FL_: Flexion/extension moment arm of LU around MCP joint
*a*_FDP_MCP_FL_: Flexion/extension moment arm of FDP around MCP joint
*a*_RI_MCP_AD_: Adduction/abduction moment arm of RI around MCP joint
*a*_UI_MCP_AD_: Adduction/abduction moment arm of UI around MCP joint
*a*_LU_MCP_AD_: Adduction/abduction moment arm of LU around MCP joint
*θ*_1_, *θ*_2_, *θ*_3_, *θ*_4_: Angles of phalanx segments with respect to the *x* axis of the global coordinate system
*l*_1_, *l*_2_, *l*_3_: Phalangeal lengths
*P*_*x*_, *P*_*y*_, *P*_*z*_: Measured fingertip forces
*k*_*i*_: Individual tendon component stiffness
*l*_*i*_: Length of the individual tendon component
*l*_*i*0_: Slack length of the individual tendon component
*s*_*i*_: Displacement of the tendon component
*p*_*jk*_: The *j*th coordinate of the *k*th node of the tendon network
*K*_*i*_: Stiffness determined by the material characteristic of the tendon
Π: Total system potential energy
*U*: Strain energy stored in the system
*V*: Potential energy of the external forces.
